# The value of Gd-EOB-DTPA-enhanced MRI in the differential diagnosis of HCC with hyperintensity on HBP and FNH

**DOI:** 10.1097/MD.0000000000043106

**Published:** 2025-06-27

**Authors:** Xin-hui Zhuang, Dong-ying Su, Miao-er Li, Jinzhan Su, Shu-feng Fan, Fang Wu

**Affiliations:** a Department of Radiology, The Second Affiliated Hospital of Zhejiang Chinese Medical University, Hangzhou, China.

**Keywords:** hepatobiliary phase, hepatocellular carcinoma, hyperintensity, nomogram diagnostic model

## Abstract

The aim was to investigate the differential diagnostic potential of gadolinium-ethoxybenzyl-diethylenetriamine pentaacetic acid (Gd-EOB-DTPA)-enhanced magnetic resonance imaging (MRI) features and establish a nomogram model for distinguishing hepatocellular carcinoma (HCC) from focal nodular hyperplasia (FNH) presenting with hyperintensity on the hepatobiliary phase (HBP). This retrospective study enrolled 80 patients with pathologically confirmed HCC or FNH who underwent Gd-EOB-DTPA-enhanced MRI between January 2017 and December 2020. All lesions exhibited hyperintensity on HBP. Morphological characteristics, signal patterns, and apparent diffusion coefficient (ADC) values were analyzed. Univariate and multivariate logistic regression analyses were performed to identify independent predictors of HCC, adjusting for age and sex. A diagnostic nomogram was subsequently constructed. After adjusting for age and sex, the study showed that nodule-in-nodule hyperintensity on HPB (odds ratio [OR] = 36.46, 95% confidence interval [CI]: 4.01–331.13), an ADC value ≤1.087 × 10^−3^ mm^2^/s (OR = 0.004, 95% CI: 0.00–0.06), and the absence of a central scar (OR = 0.04, 95% CI: 0.003–0.40) were independent predictors of HCC. The nomogram incorporating these predictors demonstrated excellent diagnostic performance, with an area under the receiver operating characteristic curve of 0.933 (95% CI: 0.874–0.991). The calibration curve showed optimal agreement between predicted and observed probabilities. Gd-EOB-DTPA-enhanced MRI characteristics combined with ADC values enable reliable differentiation between HBP-hyperintense HCC and FNH. The proposed nomogram model provides a clinically applicable tool for improving diagnostic accuracy in challenging cases.

## 1. Introduction

Hepatocellular carcinoma (HCC), the most prevalent primary hepatic malignancy and the fifth leading global and the third highest mortality rate cancer, has demonstrated a rising incidence in recent decades.^[1]^ Despite advancements in therapeutic strategies, the 5-year survival rate for HCC patients remains dismal (<20%), underscoring the critical importance of early detection to improve clinical outcomes, the 5-year survival rate for HCC patients who received early treatment is significantly higher. Gadolinium-ethoxybenzyl-diethylenetriamine pentaacetic acid (Gd-EOB-DTPA), a hepatobiliary-specific contrast agent, which is currently used for diagnosing liver lesions, has become an important diagnostic tool for HCC through the specific uptake of contrast agents on hepatobiliary phase (HBP), with a typical manifestation of hypointensity on HBP.^[2]^ However, emerging evidence reveals a diagnostically challenging subgroup of HCCs, which demonstrates atypical iso-/hyperintensity on HBP. These “HBP-hyperintense HCCs” are frequently misclassified as benign entities, leading to delayed interventions and poorer prognoses.^[3,4]^ Focal nodular hyperplasia (FNH), the second most common benign hepatic tumor, represents a key mimic of HBP-hyperintense HCCs.^[5]^ Composed of hyperplastic but functionally normal hepatocytes, FNH characteristically shows intense homogeneous Gd-EOB-DTPA uptake on HBP.^[6]^ While FNH generally requires no treatment, the starkly divergent management pathways between these entities necessitate precise differentiation.^[7]^ Current diagnostic dilemmas arise when both HCC and FNH exhibit overlapping HBP hyperintensity—a scenario inadequately addressed by existing imaging criteria. This study aims to address this critical knowledge gap by systematically comparing clinical, laboratory, and multimodal magnetic resonance imaging (MRI) features (including diffusion-weighted imaging and morphological characteristics) between pathologically proven HBP-hyperintense HCCs and FNHs. Furthermore, we develop a nomogram-based diagnostic model to empower clinicians in resolving this clinically consequential diagnostic challenge.

## 2. Methods

### 2.1. Materials and methods

This retrospective cohort study received approval from the Institutional Review Board of the Second Affiliated Hospital of Zhejiang Chinese Medical University (Ethics No: 2024-LWV-001-01) with waiver of informed consent due to the anonymized nature of retrospectively analyzed data.

### 2.2. Study population

Adult patients (≥18 years) undergoing Gd-EOB-DTPA-enhanced MRI between January 2017 and December 2020 were retrospectively collected. The inclusion criteria included: (1) Lesions showed hyperintensity on HBP; (2) histopathological confirmation of HCC or FNH via surgical resection or ultrasound-guided biopsy; and (3) complete clinical records and DICOM-format imaging data.

Exclusion criteria include are as follows: (1) evidence of hepatic failure, (2) history of pre-MRI locoregional therapy (transarterial chemoembolization, ablation, or radiotherapy), (3) nondiagnostic MRI quality (motion artifacts or incomplete sequences), and (4) missing critical clinical variables.

**Figure 1.**
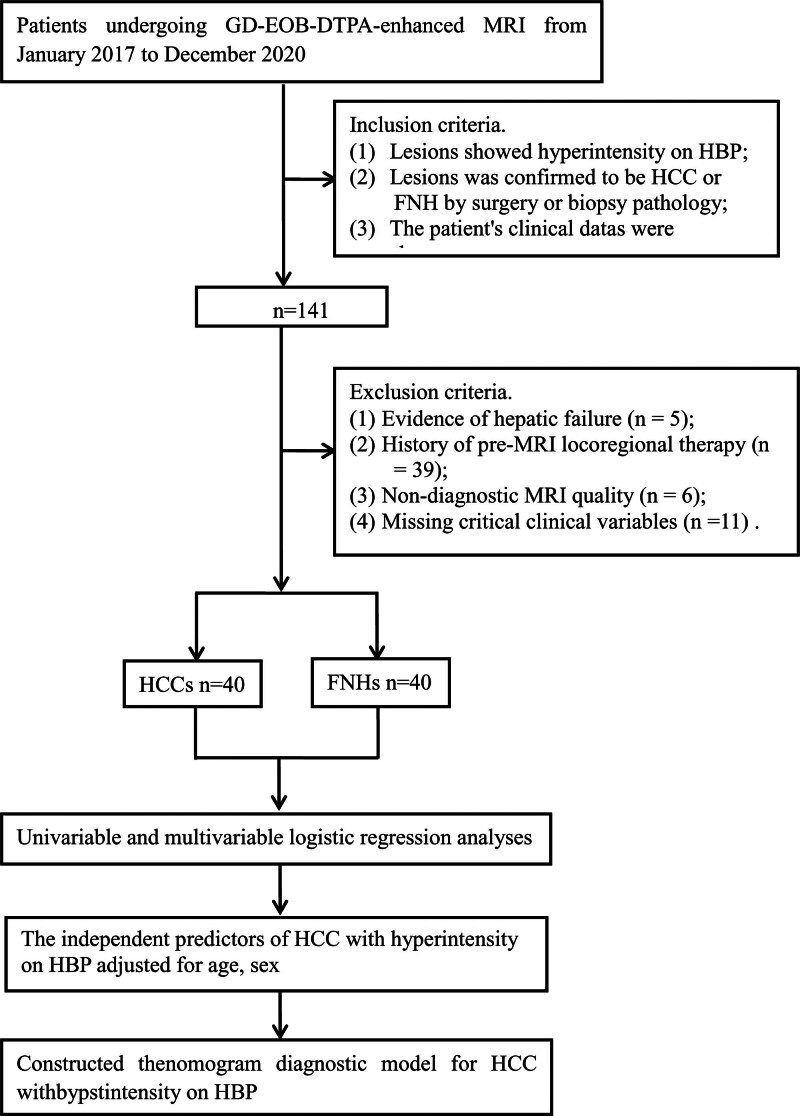
Study flow diagram. FNH = focal nodular hyperplasia, Gd-EOB-DTPA = gadolinium-ethoxybenzyl-diethylenetriamine pentaacetic acid, HBP = hepatobiliary phase, HCC = hepatocellular carcinoma, MRI = magnetic resonance imaging.

From an initial screening pool of 141 eligible patients, of which 5 patients with evidence of hepatic failure, 39 patients had received prior liver-directed therapy before MRI examination, 6 patients with nondiagnostic MRI, and 11 patients with incomplete clinical data were excluded (Fig. 1). The final cohort comprised 80 patients with histologically verified diagnoses, including 40 HCC, 30 males and 10 females, with a mean age of 51 ± 10 years; 40 FNH cases, 19 males and 21 females, with a mean age of 46 ± 15 years.

### 2.3. MR imaging and image analysis

A Siemens Avanto 1.5T MR scanner with 8-channel phased array body coils was used, and the scanning range was from the diaphragm top to the lower edge of the liver. Scan parameters, T2-weighted imaging: repetition time (TR) 4528.39 ms, echo time (TE) 85.0 ms, matrix 512 × 512, layer thickness 8 mm, field of view (FOV) 35 cm × 35 cm; diffusion weighted imaging (DWI): TR 1900 ms, TE 81.0 ms, matrix 128 × 128, layer thickness 7 mm, FOV 35 cm × 35 cm, b values of 50 and 600 s/mm, respectively. All patients underwent liver acquisition with volume acceleration prescan and Gd-EOB-DTPA multiphase dynamic enhanced scan. TR 5.7 ms, TE 2.6 ms, inversion time 5.0 ms, flip angle 15°, matrix 320 × 256, slice thickness 2.5 mm, no spacing scan, FOV 40 cm × 32 cm. After prescanning, contrast medium was injected into the peripheral vein in a bolus at a dose of 0.025 mmol/kg at a flow rate of 1.5 mL/s. After injection of contrast medium, enhanced arterial phase, portal phase, equilibrium phase and HBP scans were performed at 30 seconds, 60 seconds, 170 seconds, and 20 minutes, respectively.

**Figure 2.**
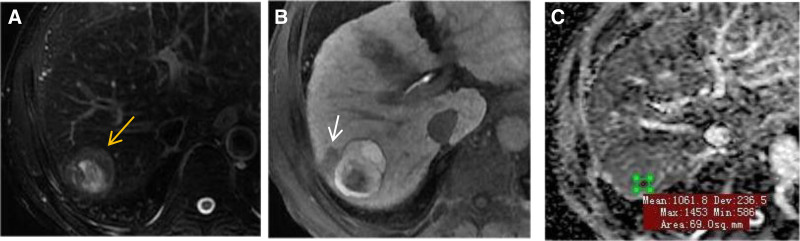
A 64-year-old male with a nodular lesion in the right lobe of the liver, without a central scar (A). Showed nodule-in-nodule hyperintensity on HBP (B), and peritumoral hypointensity on the HBP (arrow in B). The ADC value was 1.062 × 10^−3^mm^2^/s (C). The diagnosis was HCC according to the nomogram diagnostic model in this study, and the pathology was moderately differentiated hepatocellular carcinoma. ADC = apparent diffusion coefficient, HBP = hepatobiliary phase, HCC = hepatocellular carcinoma.

Two fellowship-trained abdominal radiologists (Reader A: 5 years’ experience; Reader B: 10 years’ experience) independently evaluated all MRI studies on a dedicated postprocessing workstation (GE AW 4.6, GE Healthcare, Milwaukee), blinded to pathological diagnoses and clinical parameters. Discrepancies were resolved through consensus review with a third senior radiologist (15 years’ experience). The diameter, shape, boundary, central scar, fat, capsule enhancement, signal intensity (SI) of lesions on each sequence, peritumoral hypointensity on the HBP, types of hyperintensity on the HBP (homogeneous hyperintensity, inhomogeneous hyperintensity, nodule-in-nodule hyperintensity, and ring-like hyperintensity), apparent diffusion coefficient (ADC) value were analyzed and recorded. Lesions were characterized according to the following standardized criteria:

Morphological features are as follows: (1) Diameter: Maximal axial dimension measured on the sequence demonstrating clearest lesion-liver interface. (2) Shape: Categorized as regular (oval/spherical) or irregular (lobulated/infiltrative). (3) Boundary: Clear (sharp demarcation from parenchyma) vs. indistinct. (4) Central scar: Defined as stellate T2-hyperintense core showing delayed enhancement and HBP hypointensity (Fig. 2A). (5) Fat content: Confirmed by >10% signal drop on opposed-phase T1-weighted imaging versus in-phase. (6) Capsule enhancement: Complete or partial enhancing rim persisting through portal venous and delayed phases.

Signal intensity assessment is as follows: (1) relative SI: (a) hypointense: SI <adjacent liver parenchyma; (b) Hyperintense: SI >adjacent liver parenchyma. (2) Peritumoral hypointensity on the HBP: Defined as irregular, wedge-shaped hypointensity shadows around tumors, with signals lower than those in surrounding liver tissue. (3) HBP hyperintensity subtyping: (a) Homogeneous: Uniform hyperintensity relative to background liver; (b) Inheterogeneous: Nonuniform hyperintensity with internal septations or mosaic architecture; (c) Nodule-in-nodule: Hyperintensity nodules on the HBP with internal smaller hypointensity nodules (Fig. 2B); (d) Ring-like hyperintensity on HBP: Smooth circular hyperintensity at the edge of tumors on HBP (Fig. 2B).

ADC values were measured on a GEAW4.6 workstation. Three circular region of interests (15–30 mm²) were placed in solid tumor components avoiding necrosis/cystic areas. Mean ADC (×10⁻³ mm²/s) was calculated from the triplicate measurements.

### 2.4. Statistical analysis

Data analysis was performed using IBM SPSS Statistics 26.0 (Armonk, NY) for descriptive statistics and MedCalc 15.0 (Ostend, Belgium) for diagnostic accuracy assessments, with significance set at 2-tailed *P* <.05. Categorical variables were tested using χ²/Fisher’s exact tests and continuous variables by Student *t* test/Mann–Whitney *U* test, incorporating normality assessment via Shapiro–Wilk testing. The cutoff of ADC value for distinguishing HCC with hyperintensity on HBP from FNH was based on the Youden index, while univariable screening (*P* < .05) and multivariable logistic regression with backward elimination identified independent predictors, adjusted for age, sex, and cirrhosis status after confirming the absence of multicollinearity (variance inflation factor < 5). A diagnostic nomogram was developed using R 4.2.1 and internally validated through 1000 bootstrap resamples.

## 3. Results

### 3.1. Clinical features

There were statistical differences between the HCC and the FNH in gender, alpha-fetoprotein , and CA125 (*P* < .05), but no statistical difference between the 2 groups in age (Table 1).

**Table 1:** Clinical and laboratory characteristics of HCC and FNH.

	HCC (n = 40)	FNH (n = 40)	T/χ²	*P*
Gender				
Male	30 (75.00)	19 (47.50)	6.373	.012*
Female	10 (25.00)	21 (52.50)		
Age (year)	51 ± 10	46 ± 15	−1.853	.068
AFP (ng/mL)				
>20	16 (40.00)	3 (7.50)	9.940	.002*
≤20	24 (60.00)	37 (92.50)		
CA125 (u/mL)				
>35	16 (40.00)	5 (12.50)	6.457	.011*
≤35	24 (60.00)	35 (87.50)		

AFP = alpha-fetoprotein, FNH = focal nodular hyperplasia, HCC = hepatocellular carcinoma.

*Indicates a statistically significant difference at *P* < .05.

### 3.2. Univariate analysis

Among the imaging features, there were statistical differences between HCC and FNH in terms of boundary, central scar, capsule enhancement, T2-weighted imaging signal, washout, DWI, ADC values, periumoral hypointensity on the HBP and nodule-in-nodule hyperintensity on the HBP (*P* < .05) (Table 2), among which the ADC cutoff for differential diagnosis of HCC and FNH was 1.087 × 10^−3^ mm^2^/s.

**Table 2:** Image features of HCC and FNH.

Features	HCC (n = 40)	FNH (n = 40)	T/χ^2^	*P*
Shape				
Regular	27 (67.50)	22 (55.00)	1.32	.25
Irregular	13 (32.50)	18 (45.00)		
Boundary				
Clear	22 (55.00)	32 (80.00)	5.70	.02*
Unclear	18 (45.00)	8 (20.00)		
Central scar	3 (7.50)	12 (30.00)	5.25	.02*
Fat content	8 (20.00)	2 (5.00)	2.86	.09
Diameter (cm)	2.87 (1.57–4.75)	2.72 (1.80–4.88)	−0.35	.73
Capsule enhancement	20 (50.00)	8 (2.00)	7.91	.005*
T1WI hypointense	27 (67.50)	34 (85.00)	3.38	.07
T2WI hyperintense	3 (82.50)	25 (62.50)	4.01	.05*
DWI hyperintense	36 (90.00)	27 (67.50)	4.78	.03*
APHE	33 (82.50)	38 (95.00)	2.00	.16
Wash out	16 (40.00)	6 (15.00)	6.27	.01*
ADC value (×10^−3^ mm^2^/s)	0.998 (0.877, 1.141)	1.480 (1.238, 1.766)	−5.50	<.001*
Peritumoral hypointensity on the HBP	18	9	4.53	.03*
Homogeneous hyperintensity on HBP	16 (40.00)	8 (20.00)	0.56	.46
Inhomogeneous hyperintensity on HBP	27 (67.50)	26 (65.00)	0.06	.81
Ring-like hyperintensity on HBP	16 (40.00)	8 (20.00)	2.92	.05
Nodule-in-nodule hyperintensity on HBP	18 (45.00)	5 (12.50)	10.31	.001*

ADC = apparent diffusion coefficient, APHE = arterial phase hyperenhancement, DWI = diffusion weighted imaging, FNH = focal nodular hyperplasia, HBP = hepatobiliary phase, HCC = hepatocellular carcinoma, T2WI = T2-weighted imaging.

*Indicates a statistically significant difference at *P* < .05.

### 3.3. Multivariate analysis and diagnostic performance

Variables demonstrating statistical significance (*P* < .05) in univariate analysis were incorporated into multivariate logistic regression. After adjustment for age and sex as potential confounders, 3 independent predictors of HBP-hyperintense HCC were identified: ADC value ≤1.087 × 10^−3^ mm^2^/s (odds ratio [OR] = 0.005; *P* < .001), absence of central scar (OR = 0.03; *P* = .005), and nodule-in-nodule hyperintensity (OR = 59.67; *P *= .002) (Tables 3 and 4). All variance inflation factors were <2.0, confirming negligible multicollinearity. Diagnostic performance metrics of these predictors, including sensitivity, specificity, and accuracy, are comprehensively detailed in Table 5.

**Table 3:** Multivariate analysis results.

Features	B value	OR	95% CI	Wald	*P* value
Gender	1.54	4.68	0.45–48.85	1.66	.20
Capsule enhancement	1.33	3.76	0.49–28.87	1.62	.20
Wash out	−0.60	0.55	0.03–9.40	0.17	.68
AFP	0.47	1.60	0.09–27.58	0.11	.75
CA125	2.27	9.69	0.78–120.84	3.11	.08
Boundary	−1.32	0.27	0.03–2.62	1.28	.28
T2WI	1.44	4.21	0.33–53.11	1.24	.27
DWI	1.51	4.54	0.42–48.98	1.56	.21
ADC value	−5.27	0.005	0.00–0.27	6.87	.009*
Nodule-in-nodule hyperintensity on HBP	3.94	51.21	2.05–1279.79	5.75	.02*
Peritumoral hypointensity on HBP	2.95	19.13	1.29–284.30	4.59	.03*
Central scar	−3.21	0.04	0.002–0.85	4.27	.04*
Constant	2.12	8.37	/	0.47	.49

ADC = apparent diffusion coefficient, AFP = alpha-fetoprotein, CI = confidence interval, DWI = diffusion weighted imaging, HBP = hepatobiliary phase, OR = odds ratio, T2WI = T2-weighted imaging.

*Indicates a statistically significant difference at *P* < .05.

**Table 4:** Features included in the regression equation after adjusting for age and sex.

Features	B value	OR	95% CI	Wald	*P* value
ADC value	−5.51	0.004	0.00–0.06	16.14	<.001*
Nodule-in-nodule hyperintensity on HBP	3.59	36.46	4.01–331.13	10.21	.001*
Central scar	−3.30	0.04	0.003–0.40	7.41	.006*
Constant	6.43	618.15	/	15.46	<.001*

ADC = apparent diffusion coefficient, CI = confidence interval, HBP = hepatobiliary phase, OR = odds ratio.

*Indicates a statistically significant difference at *P* < .05.

**Table 5:** Diagnostic value of various features in HCC with hyperintensity on HBP.

	AUC	95% CI	Sensitivity (%)	Specificity (%)	Accuracy (%)
No central scar	0.613	0.497–0.719	92.50	30.00	61.25
ADC value ≤ 1.087 × 10^−3^ mm^2^/s	0.858	0.761–0.926	75.00	90.00	88.75
Nodule-in-nodule hyperintensity on HBP	0.663	0.548–0.764	45.00	87.50	66.25
ADC + No central scar + nodule-in-nodule hyperintensity	0.933	0.874–0.991	90.00	92.50	91.30

ADC = apparent diffusion coefficient, AUC = area under the receiver operating characteristic curve, CI = confidence interval, HBP = hepatobiliary phase, HCC = hepatocellular carcinoma.

### 3.4. Construction of nomogram diagnostic model

**Figure 3.**
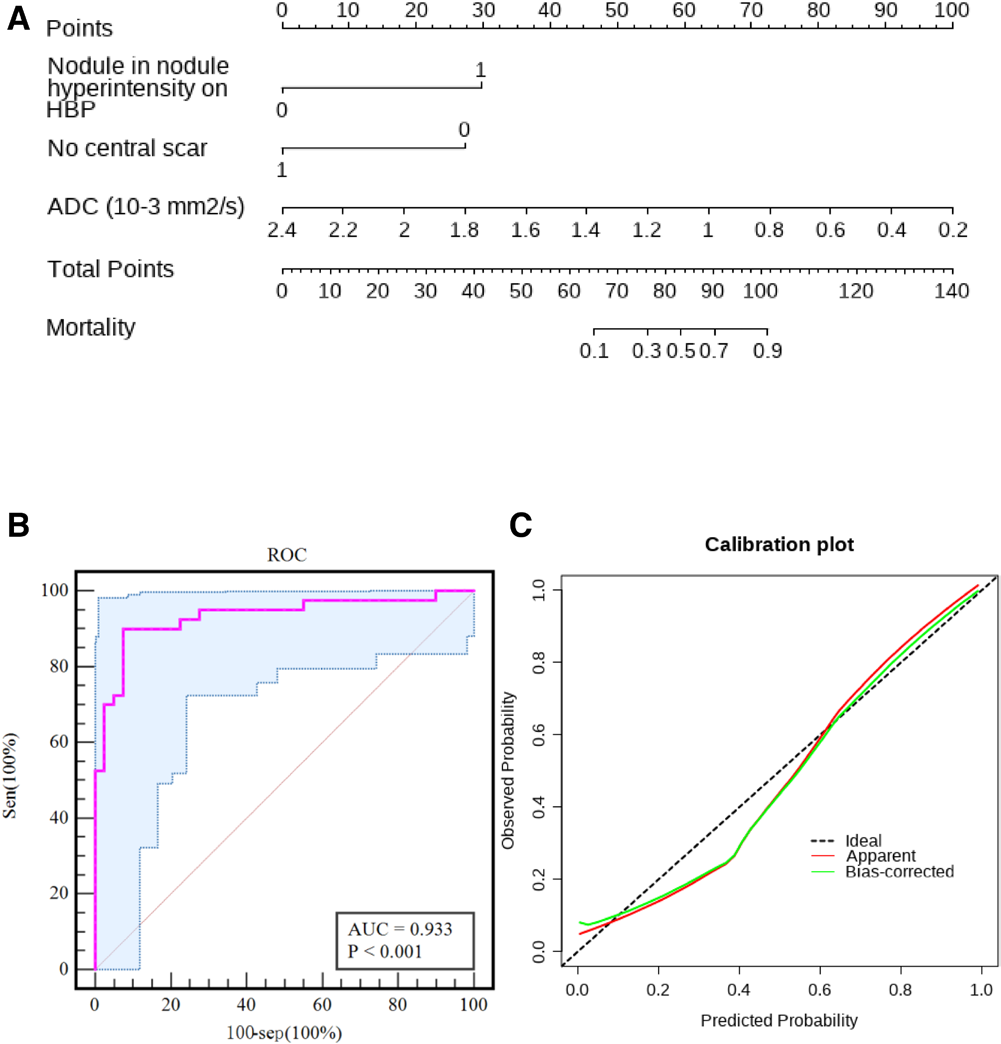
The nomogram diagnostic model, correction curve, and ROC curve of Gd-EOB-DTPA-enhanced MRI for the differential diagnosis of HCC with hyperintensity on HBP and FNH. (A) The nomogram diagnostic model. Among them, nodule-in-nodule hyperintensity on HBP 1: present, 0: absent; peritumoral hypointensity on HBP 1: present;0: absent; central scar 1: present, 0: absent. (B) Correction curve with the C index is 0.933. (C) ROC curve, AUC value is 0.933. ADC = apparent diffusion coefficient, AUC = area under the ROC curve, FNH = focal nodular hyperplasia, Gd-EOB-DTPA = gadolinium-ethoxybenzyl-diethylenetriamine pentaacetic acid, HBP = hepatobiliary phase, ROC= receiver operating characteristic.

The nomogram diagnostic model(Fig. 3A) achieving 90.0% sensitivity, 92.5% specificity, and 91.3% overall accuracy for diagnosing HBP-hyperintense HCC. Calibration analysis revealed excellent agreement between predicted and observed probabilities (C value of 0.933, *P* = .412) (Fig. 3B). Bootstrap internal validation (1000 resamples) demonstrated preserved discriminative capacity (optimism-corrected area under the receiver operating characteristic curve = 0.933, 95% confidence interval: 0.874–0.991) (Fig. 3C), confirming model generalizability.

## 4. Discussion

The hepatobiliary-specific contrast agent Gd-EOB-DTPA is actively transported into functional hepatocytes via the OATP1B3 transporter, enabling precise lesion characterization during the HBP.^[8]^ While FNH typically demonstrates homogeneous hyperintensity on HBP due to preserved OATP1B3 expression, conventional HCCs exhibit hypointensity owing to transporter downregulation. Paradoxically, some type of HCCs retain partial OATP1B3 functionality, manifesting hyperintensity on HBP that mimics FNH a diagnostic dilemma with critical prognostic implications, as these lesions often represent early-stage, well-differentiated tumors amenable to curative therapies.^[5,9]^ Therefore, we believe that early diagnosis of this type of HCCs is very important.

Our analysis of 80 histologically confirmed cases delineated that nodule-in-nodule hyperintensity on HBP, no central scar, and ADC value ≤1.807 × 10^−3^ mm^2^/s were independent diagnostic factors for HCC with hyperintensity on HBP after adjusting for age and sex.

While DWI demonstrates clinical utility in distinguishing malignant from benign hepatic lesions, its interpretation requires nuanced analysis. The hypercellular architecture of HCC restricts water molecule diffusion, typically manifesting as hyperintensity on DWI.^[10]^ However, diagnostic specificity is compromised by overlapping DWI hyperintensity in hypervascular benign entities (e.g., FNH, hepatic adenoma).To overcome this limitation, we employed ADC quantification—a robust biomarker reflecting the magnitude of diffusion restriction. Consistent with prior investigations,^[11,12]^ our cohort confirmed significantly lower ADC values in HCC compared to benign lesions. Notably, Kitao et al.^[13]^ specifically documented reduced ADC values in HBP-hyperintense HCC relative to FNH, a finding replicated in our study. Multivariable analysis established ADC ≤1.087 × 10⁻³ mm²/s as an independent HCC discriminator, achieving 90.0% specificity and 88.75% overall accuracy. This cutoff optimally balances sensitivity and specificity, addressing DWI’s inherent limitations in lesion characterization.

The nodule-in-nodule configuration is an established imaging hallmark of HCC, pathologically correlating with intratumoral heterogeneity arising from divergent differentiation states.^[14,15]^ While conventional MRI sequences demonstrate this feature through arterial phase hyperenhancement and subsequent washout in hypodifferentiated subnodules,^[16]^ our study extends its diagnostic relevance to the HBP. We identified HBP nodule-in-nodule hyperintensity characterized by hypointense subnodules (OATP1B3-deficient) embedded within hyperintense matrix (OATP1B3-retained) as an independent HCC predictor (OR = 36.46). This pattern reflects the multistep hepatocarcinogenesis from dysplastic nodules to overt HCC, where progressive OATP1B3 downregulation in dedifferentiated foci creates differential Gd-EOB-DTPA uptake.^[13]^ The detection of such architectural complexity on HBP may signify early malignant transformation, underscoring its critical role in timely diagnosis.

Central scars, pathognomonic for FNH (prevalence > 50%),^[17]^ exhibit a distinctive triphasic composition: radially organized fibrotic tissue, aberrant vasculature, and noncommunicating bile ductules. These elements collectively manifest as stellate hypointensity on HBP, surrounded by hyperintense parenchyma with preserved OATP1B3 function.^[5]^ Conversely, HCCs rarely develop true central scars—a dichotomy exploited by our model, where scar absence emerged as a potent HCC indicator (OR = 0.04). This finding aligns with established pathophysiological principles: malignant hepatocytes lose architectural organization capacity essential for scar formation.

We established a nomogram diagnostic model based on the independent diagnostic factors of HCC with hyperintensity on HBP. Each feature was visualized in the form of assignment, which can intuitively display the diagnostic value of each image feature on HCC with hyperintensity on HBP. The sensitivity, specificity, and accuracy of the nomogram model in this study for diagnosing HCC with hyperintensity on HBP were 90.0%, 92.5%, and 91.3%, respectively. The calibration curve showed that the nomogram model was in good agreement with the actual probability in distinguishing HCC and FNH.

There are some limitations in our study. First, despite rigorous inclusion criteria, the rarity of HBP-hyperintense HCC necessitates multicenter validation. Secondly, previous studies have suggested that due to the low dose of Gd-EOB-DTPA, it may have an impact on the observation of arterial phase hyperenhancement and washout, further studies are needed to explore the impact of contrast agent dosing on imaging manifestations of HCC.

## 5. Conclusion

Gd-EOB-DTPA-enhanced MRI coupled with the proposed nomogram integrating nodule-in-nodule morphology, ADC quantification, and scar assessment provides a clinically robust framework for distinguishing HBP-hyperintense HCC from FNH. This approach addresses a critical unmet need in HCC management, particularly for early-stage lesions where timely intervention maximizes survival benefits.

## Author contributions

**Formal analysis:** Xin-hui Zhuang, Miao-er Li.

**Methodology:** Xin-hui Zhuang, Dong-ying Su.

**Data curation:** Dong-ying Su, Fang Wu.

**Conceptualization:** Miao-er Li, Fang Wu.

**Investigation:** Jinzhan Su, Shu-feng Fan.

## References

[1] European Association for the Study of the Liver. EASL Clinical Practice Guidelines: management of hepatocellular carcinoma. J Hepatol. 2018;69:182–236. 29628281 10.1016/j.jhep.2018.03.019

[2] Wang YC Chou CT Lin CP Chen YL Chen YF Chen RC. The value of Gd-EOB-DTPA-enhanced MR imaging in characterizing cirrhotic nodules with atypical enhancement on Gd-DTPA-enhanced MR images. PLoS One. 2017;12:e0174594. 28355258 10.1371/journal.pone.0174594 PMC5371364

[3] Fujita N Nishie A Asayama Y. Hyperintense liver masses at hepatobiliary phase gadoxetic acid-enhanced MRI: imaging appearances and clinical importance. Radiographics. 2020;40:72–94. 31834849 10.1148/rg.2020190037

[4] Tsuboyama T Onishi H Kim T. Hepatocellular carcinoma: hepatocyte-selective enhancement at gadoxetic acid-enhanced MR imaging—correlation with expression of sinusoidal and canalicular transporters and bile accumulation. Radiology. 2010;255:824–33. 20501720 10.1148/radiol.10091557

[5] Yoneda N Matsui O Kitao A. Benign hepatocellular nodules: hepatobiliary phase of gadoxetic acid-enhanced MR imaging based on molecular background. Radiographics. 2016;36:2010–27. 27740898 10.1148/rg.2016160037

[6] Kim TH Woo S Ebrahimzadeh S McInnes M Gerst SR Do RK. Hepatic adenoma subtypes on hepatobiliary phase of gadoxetic acid-enhanced MRI: systematic review and meta-analysis. AJR Am J Roentgenol. 2022;220:28–38. 35920706 10.2214/AJR.22.27989 PMC11759479

[7] Bilreiro C Soler JC Ayuso JR Caseiro-Alves F Ayuso C. Diagnostic value of morphological enhancement patterns in the hepatobiliary phase of gadoxetic acid-enhanced MRI to distinguish focal nodular hyperplasia from hepatocellular adenoma. Radiol Med. 2021;126:1379–87. 34287759 10.1007/s11547-021-01403-2

[8] Kitao A Zen Y Matsui O. Hepatocellular carcinoma:signal intensity at gadoxetic acid–enhanced MR imaging—correlation with molecular transporters and histopathologic features. Radiology. 2010;256:817–26. 20663969 10.1148/radiol.10092214

[9] Kitao A Matsui O Yoneda N. Hypervascular hepatocellular carcinoma: correlation between biologic features and signal intensity on gadoxetic acid-enhanced MR images. Radiology. 2012;265:780–9. 23175543 10.1148/radiol.12120226 PMC5375624

[10] Chen ML Zhang XY Qi LP Shi QL Chen B Sun YS. Diffusion-weighted images (DWI) without ADC values in assessment of small focal nodules in cirrhotic liver. Chin J Cancer Res. 2014;26:38–47. 24653625 10.3978/j.issn.1000-9604.2014.01.07 PMC3937748

[11] Bilreiro C Soler JC Ayuso JR Caseiro-Alves F Ayuso C. Non-Hypervascular hypointense nodules at gadoxetic acid MRI: hepatocellular carcinoma risk assessment with emphasis on the role of diffusion-weighted imaging. J Gastrointest Cancer. 2018;49:302–10. 28547117 10.1007/s12029-017-9952-7

[12] Inchingolo R De Gaetano AM Curione D. Role of diffusion-weighted imaging, apparent diffusion coefficient and correlation with hepatobiliary phase findings in the differentiation of hepatocellular carcinoma from dysplastic nodules in cirrhotic liver. Eur Radiol. 2015;25:1087–96. 25430005 10.1007/s00330-014-3500-7

[13] Kitao A Matsui O Yoneda N. Differentiation between hepatocellular carcinoma showing hyperintensity on the hepatobiliary phase of gadoxetic acid-enhanced MRI and focal nodular hyperplasia by CT and MRI. AJR Am J Roentgenol. 2018;211:347–57. 29708786 10.2214/AJR.17.19341

[14] Xinming Z. Clinical application and prospects of Gd-EOB-DTPA enhanced MRI. Chin J Radiol. 2019;53:1025–8.

[15] Chartampilas E Rafailidis V Georgopoulou V Kalarakis G Hatzidakis A Prassopoulos P. Current imaging diagnosis of hepatocellular carcinoma. Cancers (Basel). 2022;14:3997. 36010991 10.3390/cancers14163997 PMC9406360

[16] Choi JY Lee JM Sirlin CB. CT and MR imaging diagnosis and staging of hepatocellular carcinoma: part II. Extracellular agents, hepatobiliary agents, and ancillary imaging features. Radiology. 2014;273:30–50. 25247563 10.1148/radiol.14132362 PMC4263770

[17] Guo YF Li WJ Cai WL Zhang Y Fang YJ Hong GB. Diagnostic value of gadoxetic acid-enhanced MR imaging to distinguish HCA and its subtype from FNH: a systematic review. Int J Med Sci. 2017;14:668–74. 28824299 10.7150/ijms.17865 PMC5562118

