# The value of Gd-EOB-DTPA-enhanced MRI in the differential diagnosis of HCC with hyperintensity on HBP and FNH: Erratum

**DOI:** 10.1097/MD.0000000000050268

**Published:** 2026-08-14

**Authors:** Xin-Hui Zhuang, Dong-Ying Su, Miao-Er Li, Jinzhan Su, Shu-Feng Fan, Fang Wu

In the article “The value of Gd-EOB-DTPA-enhanced MRI in the differential diagnosis of HCC with hyperintensity on HBP and FNH,”^[1]^ which appears in Volume 104, Issue 26 of *Medicine*, the following changes have been made to the online version of the article:

An additional affiliation “The First Affiliated Hospital, Zhejiang University School of Medicine, Hangzhou, China,” has been added to the second author “Dong-ying Su.”

Section 2.1. has been updated for the change in institution and ethical approval number from “This retrospective cohort study received approval from the Institutional Review Board of the Second Affiliated Hospital of Zhejiang Chinese Medical University (Ethics No: 2024-LWV- 001-01) with waiver of informed consent due to the anonymized nature of retrospectively analyzed data.” To “This retrospective cohort study received approval from the Institutional Review Board of The First Affiliated Hospital, Zhejiang University School of Medicine (Ethics No: IIT20220327A) with waiver of informed consent due to the anonymized nature of retrospectively analyzed data.”
